# Possibilities and challenges in developing and implementing an empowerment-based school-intervention in a Swedish disadvantaged community

**DOI:** 10.1093/heapro/daz021

**Published:** 2019-03-08

**Authors:** L Jonsson, A Fröberg, P Korp, C Larsson, C Berg, E -C Lindgren

**Affiliations:** 1 Department of Food and Nutrition and Sport Science, University of Gothenburg, Gothenburg, Sweden; 2 School of Health and Welfare, Halmstad University, Halmstad, Sweden

**Keywords:** food, participation, physical activity, shared decision making, socioeconomic

## Abstract

In this paper, we describe and critically reflect on the possibilities and challenges of developing and implementing an empowerment-based school intervention regarding healthy food and physical activity (PA), involving participants from a Swedish multicultural area characterized by low socioeconomic status. The 2-year intervention was continually developed and implemented, as a result of cooperation and shared decision making among researchers and the participants. All 54 participants were seventh graders, and the intervention comprised health coaching, health promotion sessions and a Facebook group. We experienced that participants valued collaborating with peers, and that they took responsibility in codeveloping and implementing the intervention. Participants expressed feeling listened to, being treated with respect and taken seriously. However, we also experienced a number of barriers that challenged our initial intentions of aiding participation and ambition to support empowerment. Moreover, it was challenging to use structured group health coaching and to work with goal-setting in groups of participants with shared, and sometimes competing, goals, wishes and needs related to food and PA. Successful experiences from this intervention was the importance of acquiring a broad and deep understanding of the context and participants, being open to negotiating, as well as adjusting the intervention.

## INTRODUCTION

Many lifestyle interventions have been conducted among adolescents, yet such interventions have been rather unsuccessful in promoting food (Evans *et al.*, 2012) and physical activity (PA) (Borde *et al.*, 2017). Whilst researchers seldom provide opportunities for adolescents to participate in the development and implementation of such interventions, participants should have the right to express their opinions and to be heard in matters affecting their health (United Nations, 1989).

Adolescents form a group of diverse individuals with varying health-related needs, wishes and goals. They also possess ideas and suggestions for activities that could be incorporated into an intervention, to improve its appropriateness and the acceptability (Sawyer *et al.*, 2012). Consequently, it is pivotal to develop interventions based on adolescents’ goals, desires and needs in relation to food and PA habits. Although attempts have been made to include adolescents in health-related research by following participatory approaches, relatively few have involved adolescents in developing and implementing interventions focused on food and PA (Jacquez *et al.*, 2013; Frerichs *et al.*, 2016). Furthermore, a more recent study by Larsson and colleagues (Larsson *et al.*, 2018) systematically reviewed participatory interventions focusing on health and well-being with children and young people. None of the PA interventions involved the children or adolescents in the development or implementation of the interventions. However, there are some promising examples indicating that participants are positive to having a voice in the development and implementation of interventions and are able to identify barriers for PA that are easily overlooked, unfamiliar or perceived differently by adults (Caro *et al.*, 2016; Verloigne *et al.*, 2017).

We have developed and implemented an empowerment-based school intervention, exploring how to support adolescents in achieving and maintaining healthy food and PA habits (the ‘How-to-Act?’ project). Although previous studies involving youths across different phases of the research-process have reported overwhelming advantages in terms of empowerment, relatively few of these studies appear to be interventions (Jacquez *et al.*, 2013). Among available participatory-based interventions focusing on lifestyle-habits, the most common approach has been to engage youths as peer-leaders to implement pre-defined strategies with the intention of encouraging healthy lifestyle among their peers. The majority of these interventions is also obesity-prevention rather than involving a salutogenic approach (i.e. focusing on facilitating salutary factors that can actively promote health, such as PA, rather than trying to reduce pathogenic risk factors) (Frerichs *et al.*, 2016). Moreover, in the area of health promotion interventions, not necessarily empowerment-based, there have been some suggestions on step-based approaches to developing, implementing and evaluating interventions (see Fraser and Galinsky, 2010; Eldredge *et al.*, 2016 for further information). One example of an intervention that followed this step-based approach is a recent study by Lindqvist and Rutberg (Lindqvist and Rutberg, 2018), who developed an active school transportation intervention to promote children’s PA. First, they identified factors related to active school transportation (for example self-efficacy) and agents who control environmental factors (for example parents). Second, they developed the intervention by involving the children and their parents and teachers. Third, they developed a plan for evaluation and implementation by, for example, conducting focus group interviews with the children and teachers from the intervention, and planned for scaling up the intervention to include several schools.

Furthermore, we know relatively little about the processes of developing and implementing empowerment-based interventions that provides opportunities for involvement in the change-possess while simultaneously acknowledging the need for evidence-based health-information. Thus, the aim of this paper is to describe and critically reflect on the possibilities and challenges of developing and implementing an empowerment-based school intervention regarding healthy food and PA, involving participants from a Swedish multicultural area characterized by low socioeconomic status (SES).

## INTERVENTION SETTING

### Intervention school

We invited schools in the area of Angered in Gothenburg, Sweden to volunteer for their participation as an intervention school. One reason for targeting schools in Angered was their joint decision to work towards becoming health promoting a school. In a meeting with all school health service teams, including principals, we informed these key stakeholders about our intervention idea and expressed our wish to cooperate with a school where the staff supported our basic ideas for the intervention. After discussions with a few candidate schools, we agreed on collaboration with one school.

Angered was also chosen for this intervention since it is characterized by low SES, and adolescents from low SES circumstances might have less healthy food and PA habits (Hanson and Chen, 2007). Recent trends also suggest that rising socioeconomic inequality among adolescents have also exacerbated inequality in health during the last decade (Elgar *et al.*, 2015), and that it is therefore imperative to intervene in the health-affecting habits of adolescents of low SES areas. Furthermore, baseline data suggested that many participants did not meet the PA recommendations (Fröberg *et al*., 2018a), and the participants identified a number of perceived undermining factors in relation to their healthy food and PA habits (Jonsson *et al*., 2017).

Angered is a multicultural area where a high proportion (72%) of residents has a foreign background (Lundquist, 2014). It currently ranks among Sweden’s most vulnerable area, as demonstrated by its parallel social structures, religious extremism and reluctance among residents to participate in judicial processes (Nationella Operativa Avdelningen, 2017). As a consequence, residents report a relatively high level of fear of spending time outside alone (Social resursförvaltning Göteborgs Stad, 2014).

In the intervention school, the pupils had low educational achievement compared with the Swedish national average, and their parents had a lower education relative to the national average (The Swedish National Agency for Education, 2014).

### Participants

We invited all 54 (*n* = 32 girls) seventh graders (aged 12–13 years) who attended the school to participate in developing and implementing the intervention. All pupils agreed to participate in the intervention. New pupils who were transferred to the school during the 2 years of the intervention were invited to participate. During school visits, we also presented the pupils with oral information about our ambition to develop and implement the intervention in a style of cooperation and shared decision making involving us and them. It was clarified that participation was voluntary, and that they could withdraw their participation at any moment without providing any further explanation or justification.

All participants and their parents or legal guardians provided their signed, written and informed (in Swedish, Arabic and Somali) consent prior to their participation.

## INTERVENTION DESIGN

**Fig. 1:**
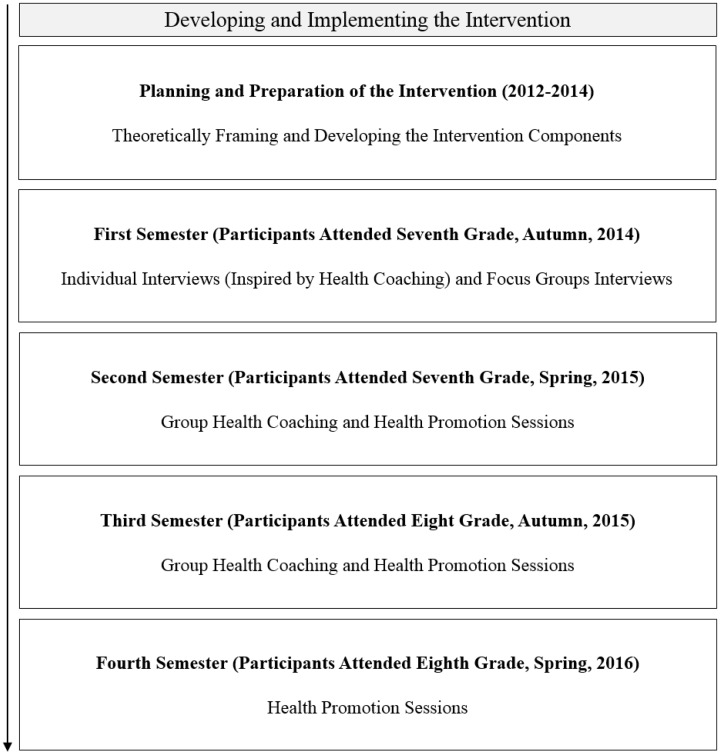
The flow of the intervention, which was planned and prepared over 2.5 years (January 2012 to June 2014), and developed and implemented in the school environment in 2014–16 as a result of cooperation and shared decision making among the authors and the participants.

Figure 1 illustrates the flow of the intervention. In 2012–14, we planned and prepared the intervention by theoretically framing and developing the intervention and its components. Thereafter, the intervention was developed and implemented in the school environment, for maximal cooperation and shared decision making among researchers and participants. We started with baseline measurements, but the evaluation of the intervention will be described elsewhere (Fröberg *et al*., 2018b) We followed the participants for two consecutive school years (2014–16) from seventh to ninth grade, covering four semesters—two semesters in seventh grade and two semesters in eighth grade (henceforth referred to as the first, second, third and fourth semester respectively), and thereafter endpoint measurements in ninth grade.

We defined empowerment as referring to possibilities for one to formulate and influence opportunities and barriers for change, and procuring motivation and belief in one’s own ability to achieving and maintaining healthy food and PA habits. Moreover, the intervention embraced the ideas of empowerment as both a goal and a process (Tengland, 2007, 2008). As a goal, the intervention aimed to facilitate self-control, knowledge, autonomy, self-esteem and self-confidence, aligned with the definition of empowerment nominated by Tengland (Tengland, 2007, 2008). Inspired by Tengland (Tengland, 2007, 2008), as a process, the aim was for the intervention to be continually developed and implemented as a result of cooperation and shared decision making, where we would support the participants by helping them to express their goals, desires and needs; would listen to their ideas; and would put these suggestions into practice. In practice, we tried to fulfil this aim by employing health coaching, as described below.

Both bottom-up and top-down approaches to health promotion have faced criticism due to the ethical tensions and dilemmas that they involve (Braunack-Mayer and Louise, 2008). However, in cultivating empowerment, the reflective equilibrium community empowerment approach allows interventions such as ours to combine the two types of approaches: to involve participants in decision-making processes and to simultaneously acknowledge the need for an intervention to be guided by health information (Braunack-Mayer and Louise, 2008).

### Health coaching

In the intervention’s approach, health coaching permeated communication during collaboration among us (the research group) and the participants. Inspired by previous research (Olsen, 2014), we defined health coaching as a process of supporting participation through the communication technique of dialogue, with the purpose of facilitating reflection, confidence in own ability and strategies for health-promoting action. We regarded participants as creative, resourceful and capable of finding unexpected solutions to fulfil their goals and to articulate their wishes and needs in relation to food and PA. Hence, health coaching was considered crucial for the empowerment process as it is emphasized that: (i) problems formulation, the problems solutions and subsequent actions should come from the participants, whereas we, as researchers, should act as facilitators and minimize our use of control (Tengland, 2007); (ii) we, as researchers, should trust in participants’ capacity and their ability to solve their own undertakings and (iii) we, as researchers, should create a climate that is characterized by empathy, authenticity and unconditional positive regard and act in a tolerant and non-judgmentally manner (Tengland, 2007).

To explore participants’ initial goals, wishes and needs, individual (*n* = 52, mean duration per interview: 20 min) and focus groups (*n* = 10, mean duration per interview: 69 min) interviews were conducted with participants during the first semester. These interviews were further supplemented by collecting written statements of participants’ individual goals for the intervention.

We also used group health coaching in a structured manner to discuss participants’ shared goals, wishes and needs as well as their resources to work towards meeting these. We anticipated that working with goal-setting strategies could be challenging, although not impossible, given that young people in these age groups are most interested in the present and engage in less thinking about the future (Sawyer *et al.*, 2012). The structured group health coaching sessions were organized according to the resource- and goal-oriented conversation technique T-GROW (Downey, 2003). While planning and preparing the intervention, we undertook a series of educational sessions, to create a shared foundation of health coaching and T-GROW. These series of educational sessions were led by a qualified coach, with extensive experience in theoretical and practical coaching processes.

In addition, a Facebook group was launched in the beginning of the intervention, with the aim of providing a forum for communication among us and the participants.

### Health promoting sessions

We put participants’ suggestions into practice by developing and implementing intervention activities that consisted of health promotion sessions. The health promotion sessions were integrated into the ordinary school schedule (i.e. they were part of the participants regular curriculum) and were generally conducted in the school environment and its surroundings (classroom, home-economics kitchen, gym, etc.). The goal was that the theme, aim and content of each health promotion session reflected the participants’ goals, wishes and needs and also our common experiences and reasonable actions for implementation. The latter meant that we reflected upon whether participants’ suggestions of activities during the health promotion sessions (theme and content) fell within the framework of the intervention and could be delivered within the given time frame and physical environment. Nevertheless, the framework of the intervention revolved around healthy food and PA, which we broadly referred to as a balanced diet rich in fruit and vegetables, low in high-calorie low-nutrient foods and sugar-sweetened beverage consumption, along with a physically active lifestyle with less sedentary behaviour.

### The role of the research group

During fieldwork, two authors (L.J. and A.F.) assumed chief responsibility for conducting the structured group health coaching sessions and the health promotion sessions, with the support of other researchers. Students studying Health Promotion at the Department of Food and Nutrition, and Sport Science, University of Gothenburg were also invited to conduct their supervised practical-work experience within the intervention; in particular, the students assisted us in cooperating with participants to develop and implement the health promotion sessions. Participants’ homeroom teachers were also invited to be involved in health promotion sessions. These homeroom teachers were informed about the shared foundation of health coaching and were provided with information regarding the theme, aim and content of the health promotion session prior to each occasion.

To enable description of and reflection on the intervention process, we documented the structured group health coaching sessions and health promotion session. The structured group health coaching session protocols consisted of 36 self-evaluation forms (*n* = 56 pages in total) where we documented expectations, experiences and general observations. The health promotion session protocols contained theme, aim, content, location, instructions and attendance rate (*n* = 52 protocols, 183 pages in total). Also documented were perceived participation, general observations and reflections (*n* = 57 protocols, 68 pages in total).

During monthly meetings the authors reviewed and summarized their observations and reflections and the participants’ expressions, to construct a framework for critically reflecting upon the lessons learned during the intervention. In an iterative process the intervention was developed and implemented in five stages as inspired by Kemmis *et al.* (Kemmis *et al.*, 2014):

1. Acting and observing: we used health coaching/structured group health coaching sessions and health promotion sessions, and observed the process;
2. Reflecting: we reviewed and summarized protocols to provide a framework to critically reflect over the process related to the health coaching/structured group health coaching sessions and the health promotion sessions;
3. Re-planning: we re-structured the intervention based on the lessons learned during the health coaching/structured group health coaching sessions and health promotion sessions;
4. Acting and observing: we used the modified health coaching/structured group health coaching sessions and/or health promotion sessions, and observed the process;
5. Reflecting: see stage (2), and so forth.

All authors participated in the reflection procedure, forming a heterogeneous constellation of collaborators with various types of expertise (food and nutrition, sports science and health promotion), who collectively possessed a broad, interdisciplinary base of knowledge. Thus the authors had various preunderstandings. More specifically, L.J. has a degree of Bachelor (BSc) in Psychology, a degree of Master (MSc) in Psychology and is currently a PhD student in Sport Science. A.F. is a Senior Lecturer in Sport Science and a Physical Education teacher. A.F.’s main research interests revolves around PA and sedentary behaviour among youth. P.K. is an Associate Professor in Sociology with a main research interest in the area of health promotion. C.L. is a nutritionist, public health scientist and a Professor in Food and Nutrition. C.L.’s research mainly revolves around dietary intake surveys and interventions regarding food habits and PA among predominately children and adolescents. C.B. is a registered dietician and a Professor in Food and Nutrition. C.B.’s main research interests are health promotion, food choice and dietary habits. E.-C.L. is a Professor in Sport Science and her research is mainly in the area of health promotion (PA, body and empowerment) in school setting and sports in children and youths.

## HOW THE INTERVENTION EVOLVED

### First and second semester

The first semester (participants attended seventh grade) began with individual and focus group interviews (Figure 1). The information produced in these interviews, together with collected written statements of participants’ goals for the intervention, guided the division of participants into groups and the initial content of the health promotion sessions in the second semester (see Table 1).

**Table 1:** Examples of the themes, contents, aims, and location of health promotion sessions across the second and fourth semester (attendance rates (per session and participant) during the health promotion sessions is shown in the table footnote).

Semester	Theme	Example of content	Aim	Location
Second semester (Spring 2015)^a^	Food, PA and health	Half-day with food-related activities (e.g. identifying the amount of added sugar in common foods) and PA (e.g. playing sports)^b^	To provide an opportunity for an engaging experience related to food and PA	Outside the school environment
		Whole day of preparation (e.g. searching online with computer tablets for recipes) and cooking of vegetarian food and exhibition concerning health and health promotion^b^	To provide an opportunity for an engaging experience related to healthy eating	Outside the school environment
	Food	Online searches with computer tablets and compilation of health-related benefits of a balanced, healthy diet as well as recommendations and guidelines^b^	To provide an opportunity to critically reflect upon and appraise health-related information	School classroom
		Preparation of healthy snacks such as smoothies containing fruits and vegetables^b^	To provide an opportunity to reflect upon daily recommendations of fruit and vegetable consumption and to learn and practice preparing healthy snacks	Home-economic kitchen
		Workshops to identify desired changes in food served at school subsequently formulated into questions and presented to representatives of the school cafeteria^b^	To provide an opportunity to identify and discuss desires for changes in the school cafeteria	School classroom
		A week of documenting whole-day dietary habits with a photo diary on smartphones	To provide an opportunity to identify and discuss food habits	In- and outside the school
	PA	Resistance training exercises focused on body-weight^b^	To provide an opportunity to learn and enact resistance training exercises not requiring any equipment	School classroom and gym
		Online searches with computer tablets and compilation of health benefits of PA as well as recommendations and guidelines (e.g. steps and minutes per day)^b^	To provide an opportunity to critically reflect upon and appraise health-related information	School classroom
		Playing sports and other physical activities (e.g. soccer, basketball, jogging or running, martial arts, brisk walking with a pedometer, dancing and swimming)	To provide opportunities to be physically active, predominately in the school’s surrounding, by inspiring, positive experiences	School classroom, school surroundings and outside the school environment
Third semester (Autumn 2015)^c^	Food and PA	Preparation and execution of a whole day of cooking vegetarian food and healthy snacks, and PA (e.g. playing sports) (group 1)	To identify and reflect upon opportunities and actions to prepare and practice activities related to healthy eating and PA, as well as to provide an opportunity for an engaging experience with healthy eating and PA	School classroom and outside the school environment
		Creation and organization of individualized food and PA programs aimed to increase fruit and vegetable consumption and decrease the consumption of energy-dense snacks and sweetened beverages, as well as to increase PA (e.g. by dancing, walking with pedometers and exercise training), during the school day (group 2)	To provide an opportunity to create and organize individualized food and PA programs and to be physical active during the school day	School classroom and outside school environment
		Ball games (group 3)	To provide an opportunity for an engaging experience with PA	Gym
Forth semester (Spring 2016)^d^	Food	Sapere workshop on exploring and increasing awareness of food-related senses and preferences^b^	To provide an opportunity to reflect upon food preferences and try different foods	School classroom
	Health	Workshop involving video clips to discuss bodies and body ideals^b^	To provide an opportunity to discuss and critically reflect upon body ideals in today’s society	School classroom
		Visiting exhibition addressing health and health promotion^b^	To provide an opportunity to learn and reflect upon different aspects of health	Outside the school environment

a Fifteen health promotion sessions, including structured group health coaching sessions (90 min per session and week), apart from two sessions (80 and 360 min, respectively); six groups (6–8 participants per group); attendance rate (range): 83% (69–89%); attendance rate per participant (range): 20–100%.

b All participants regardless of group.

c Thirteen health promotion sessions, including structured group health coaching sessions (60 min per session and week) apart from one session (360 min); four groups (group 1: *n* = 17 participants; group 2: *n* = 3 participants; group 3: *n* = 24 participants; group 4: *n* = 10 participants); attendance rate (range): 86% (80–95%); attendance rate per participant (range): 61–100%.

d Three health promotion sessions (120–180 min per session); attendance rate (range): 87% (85–89%); attendance rate per participant (range): 33–100%.

We used structured group health coaching sessions to identify common goals within each group of participants, as well as the activities they wished to focus on during the health promotion sessions (Figure 1). Through a collaborative process involving us and the participants, each group first created a regulatory framework with agreed-upon rules of conduct during the structured group health coaching sessions (for example ‘Show respect for the opinions of others’ and ‘Do not interrupt’). Although each group had created such a regulatory framework, we experienced that several participants tended to interrupt others, and a substantial part of some structured group health coaching sessions was spent focused on organizing and structuring the session, instead of the content of the dialogue. Participants also expressed disappointment in discussing goals related to food and PA, as these theoretical sessions shared features with everyday schoolwork.

We experienced them particularly appreciating practical, hands-on health promotion sessions. They were in general curious about the health promotion session and had several suggestions for activities in relation to food and PA. We further experienced that participants valued collaborating with peers, and they took responsibility in codeveloping and implementing the intervention. Generally, we experienced that groups of participants became highly influenced by the work of others. When one group engaged in activities related to food and PA (for example preparation and cooking of food, swimming), participants in other groups expressed their wishes to do a similar activity, ultimately leading to most groups having the same suggestions for activities to perform during health promotion session.

During the structured group health coaching sessions, we experienced that participants remained focused on the present and had limited interest in formulating long-term goals and working on goal-setting strategies. Those participants who we perceived as motivated to work with goal-setting strategies explicitly expressed their concern of being challenged with recalling their goals from one session to the next.

Participants suggested that, as an alternative, the structured group health coaching sessions should be replaced by practical activities, such as preparing food and playing sports. Instead of conducting structured group health coaching sessions, we agreed to end each health promotion session with brief health coaching, to reflect upon participants’ experiences and to discuss potential benefits of certain food and PA habits, in addition to strategies to implement activities promoting those habits outside the school environment. By increasingly focusing on practical, hands-on activities, the modified versions of both the health coaching component and the health promotion session improved, as the participants became increasingly involved in the activities.

### Third semester

During the third semester, the participants could choose between four groups, the contents of which reflected participants’ wishes and needs as identified during the first and second semesters.

Group 1 (*n* = 17 participants) formulated the goal to prepare and perform a whole-day session that addressed food and PA for all participants in all four groups. During the participants’ preparation, we encouraged and supported them to comply with arguments for their decisions to choose specific contents related to each activity included in the whole-day session. They agreed on practical, hands-on activities such as preparing vegetarian food, healthy snacking and doing PAs. Group 2 (*n* = 3 participants) created individualized food and PA programs, because they wished to increase their fruit and vegetables consumption and their school-day PA. Group 3 (*n* = 24 participants) focused on ball games, and group 4 (*n* = 10 participants) chose to do homework and only take part in the joint activities with all participants. For practical reasons, the homeroom teachers assumed chief responsibility for the activities in groups 3 and 4.

Compared to previous semesters, we experienced that the structured group health coaching sessions improved during this semester, possibly because more homogenous groups had been created and considerably more attention was paid to identifying shared interests within the group. We further perceived that participants in groups 1 and 2 took responsibility and cooperated with each other to plan and organize the intervention activities.

In addition to the work performed in each group, we invited a representative from a community program to participate in a workshop addressing opportunities for PA (information regarding exercise training and involvement in sport centres in Angered). The discussion was summarized by participants in groups 1 and 2, which was presented to all participants via posters displayed in the school environment.

### Fourth semester

Three health promotion sessions involving all participants were implemented during the fourth semester. Based on the participants’ requests they focused on body ideals, food (sapere) and testing a public health promotion exhibition.

In addition, we implemented a whole-day workshop at the University of Gothenburg for the school principal, teachers and other school personnel to support the school in additional tasks related to health promotion. The workshop involved the presentation of preliminary results from the intervention, an introduction to the basic principles and concepts of the intervention (empowerment and health promotion), and discussion about opportunities and challenges for sustaining the health-promotion actions in the school once the intervention had ended.

## CRITICAL REFLECTIONS AND RECOMMENDATIONS

Our aim was to continually develop and implement the intervention as a result of cooperation and shared decision making among us and the participants. The reality presented a number of barriers that challenged our goal of aiding participation and support a sense of empowerment during the development and implementation of the intervention. Other issues experienced included we experienced barriers relating to the context of the intervention (i.e. the school environment), as it was somewhat chaotic; some participants occasionally acted hostile, and during the course of the intervention, incidents occurred involving not only vandalism and fire at the school, but also physical assaults of some participants.

However, the school principal and homeroom teachers were very cooperative and shared our participatory ambitions, which was necessary for its development and implementation. Given that our empowerment goal was quite challenged when facing reality, it is reasonable to assume that without such positive treatment, we would have encountered even greater challenges during the intervention. Moreover, although the context of the intervention was challenging, we experienced that participants were curious about the intervention, as demonstrated by an eagerness to learn about the health promotion sessions and to get involved in decision-making processes. As this curiousness and willingness evolved over time, we believe that cultivating relationships and trust is pivotal to the success of interventions such as the one described in this paper.

During the course of the intervention, the participants reported a number of positive experiences such as feeling listened to, being treated with respect and taken seriously, and perceiving themselves as having had the opportunity to influence and decide on content related to the health promotion sessions. Having the opportunity to choose content related to the health promotion session made participants take responsibility, and they perceived themselves to be more collaborative with their peers during the intervention. Moreover, participants explicitly stated that practical, hand-on activities like preparing food and playing sports were increasingly engaging and enjoyable, and facilitated skill-development. By being offered opportunities to try and learn different activities related to food and PA, some participants perceived themselves as having removed some barriers to engaging in similar activities outside the intervention, such as preparing food in the home and visiting the gym during leisure time. These observations were further confirmed in focus group interviews after the intervention (Holmberg *et al*., 2018). In essence, such positive experiences might connect to some empowerment goals, as they might reflect changes in one’s self-esteem and self-confidence and thus the belief in one’s general abilities to handle-specific tasks (Tengland, 2007).

Although partly predicted, looking back with self-criticism, we realize that the intervention featured some characteristics that would collectively engender a number of challenges. For one, the intervention involved a complex process, as it was continually developed and implemented as a result of cooperation and shared decision making. As part of the complex process, much of the intervention success was due to the extent that structured group health coaching sessions worked according to our intentions. As mentioned throughout the paper, this was not always the case.

We experienced that participants seemed to live for and act in the present, and had limited interest in formulating goals and working with goal-setting strategies, both short and long terms. In addition, those participants we perceived as being motivated to set goals expressed their concern of being challenged to recall their goals from one session to the next. Another barrier that challenged the intention of working with goals was that participants tended to become influenced by the work of others, which led to most groups having the same suggestions for activities to perform during health promotion sessions.

Future, empowerment-based, health-promotion interventions might consider using structured group health coaching sessions if involving older adolescents. Capacity for goal setting increases with age (Sawyer *et al.*, 2012), and structured group health coaching sessions might be a fruitful way of identifying participants’ goals, wishes and needs when they are older adolescents. Based on our experiences, we recommend that considerable effort should be devoted to establishing frameworks of the structured group health coaching sessions, such as agreeing on the group’s rules of conduct and putting considerable effort in identifying goals, wishes and needs within each group of participants. As an alternative to structured group health coaching sessions, individual health coaching—despite being more resource-intensive—may also be worth considering in future empowerment-based health-promotion interventions. Individual health coaching creates opportunities to work with more individualized goals in relation to food and PA and adopts activities accordingly. In addition, individual health coaching may present fewer challenges by eliminating the role of group dynamics, which might engender competing interests among participants. In our intervention, such competing interests might have contributed to a sense among participants that their suggestions for activities were not fully realized, because group members were required to compromise with each other as part of their cooperation. Such competing interests constitute an ethical dilemma that needs consideration. Another ethical dilemma is to solely focusing on food and PA in an area characterized by low SES, when other health issues may require attention. However, adolescents from low SES circumstances generally have poorer food and PA habits (Hanson and Chen, 2007), which, from our perspective, founded a well-intentioned argument to focus on these two health-related habits despite the anticipated challenges. Moreover, our intent was that the participants would be provided with opportunities to critically reflect upon and appraise health-related information and recommendations in general.

A potential challenge could also be to balance the aim to promote health with the aim to aid participation and support empowerment. The framework dictated that the intervention would revolve not only around food and PA, but also *healthy* food and PA. Although the theme, aim and content of each health promotion session reflected the participants’ suggestions for activities it was also determined by our pre-set values of what we considered to be healthy food and PA.

We believe that our way of combining participants’ suggestions for activities with our pre-set values share some features with the reflective equilibrium community empowerment approach. If we had relied entirely on a bottom-up approach, where our participants would have had control over all aspects of the intervention, we probably would have found ourselves in a position of prioritizing activities that we believe would have been counter-productive. Instead, the health promotion sessions were developed and implemented based on both participants’ suggestions for activities and our pre-set values, as we acknowledge the need for the intervention to be guided by health information. On occasions where participants had suggestions for activities we considered to be less healthy, we had the opportunity to engage them in discussion to critically reflect on what constitutes healthy food and PA, and what possible options could be healthier. We acknowledge, however, that an ethical dilemma of doing so means that our intervention might have become a normative enterprise, because it recognized beneficial health-related habits and both acceptable and unacceptable risks (Massé and Williams-Jones, 2012). By the same token, it is possible that our intervention contributed to moralizing and blaming participants who did not adhere to culturally appropriate health-related habits (Massé and Williams-Jones, 2012).

The intervention was implemented during school hours, and the health promotion sessions were integrated into the school schedule. As a consequence, participants might have felt obliged to actively participate in intervention activities, because attendance is compulsory at elementary school. To handle this ethical dilemma, we continually informed them about the voluntariness of the intervention and gave them the opportunity to attend but only observe, and to choose theme groups focused on doing homework instead of health promotion session addressing food and PA. In a sense, one might consider participants who focused on doing homework during the third semester as drop-outs. However, we do not consider these participants as drop-outs for mainly by two reasons. First, it was the participants, themselves, who suggested a group that could focus on doing homework. As such, we listened to the participants suggestions and supported their suggestion into practice (i.e. adhering to our empowerment-based approach). Second, these participants were exposed to other health promoting sessions during the third semester, such as, the whole day, prepared by participants in group 1, of cooking vegetarian food and healthy snacks, and PA (for example playing sports).

## CONCLUSIONS

The intervention was developed and implemented through cooperation and shared decision making among us and the participants. We experienced that participants were generally curious about the health promotion sessions, had several suggestions for activities related to food and PA, and particularly appreciated practical, hands-on health promotion sessions. We also experienced that participants valued collaborating with their peers, and that they took responsibility in developing and implementing the intervention, such as by planning and organizing intervention activities. However, the reality presented a number of barriers that challenged our initial intentions to aid participation and support a sense of empowerment. Above all, the reality challenged our aim to use structured group health coaching sessions and to work with goal setting in groups of participants with somewhat-shared goals, wishes and needs related to food and PA. A lesson learned from this intervention is the importance of acquiring a broad and deep understanding of the targeted context and the participants of the intervention, and to be open-minded when it comes to negotiating, adjusting and reorganizing empowerment-based interventions. Moreover, our experiences suggests that it is important to: (i) recruit a school where the principal, homeroom teachers and pupils are positive from the beginning of the intervention; (ii) implement the intervention during school-hours; (iii) carefully consider how health coaching is implemented to best meet the needs of the participants, and how it suits youths’ lack of ability to set future goals and (iv) focus on implementing practical activities, rather than theoretical and/or sedentary activities.

For future research, it might be feasible to try a similar intervention approach with older adolescents who generally have a stronger future orientation.
